# High spatial and temporal resolution dynamic contrast-enhanced magnetic resonance angiography (CE-MRA) using compressed sensing with magnitude image subtraction

**DOI:** 10.1186/1532-429X-15-S1-E82

**Published:** 2013-01-30

**Authors:** Stanislas Rapacchi, Fei Han, Yutaka Natsuaki, Randall M Kroeker, Adam N Plotnik, Evan Lehrman, James Sayre, Gerhard Laub, J Paul Finn, Peng Hu

**Affiliations:** 1 Deparment of Radiological Sciences, University of California Los Angeles, Los Angeles, CA, USA; 2 Siemens Healthcare, Los Angeles, CA, USA; 3 Department of Bioengineering, David Geffen School of Medicine, University of California Los Angeles, Los Angeles, CA, USA; 4 Biomedical Physics Inter-Departmental Graduate Program, University of California Los Angeles, Los Angeles, CA, USA; 5 Department of Biostatistics, School of Public Health, University of California Los Angeles, Los Angeles, CA, USA

## Background

Due to limitations in image acquisition speed, dynamic CE-MRA typically has lower spatial resolution than conventional CE-MRA. In a breath-held dynamic CE-MRA acquisition, the subtraction of a pre-contrast mask to all post-contrast frames promotes sparsity of the resulting difference images. This "subtraction sparsity" using direct k-space complex subtraction has previously been shown to benefit parallel imaging as well as compressed sensing but suffers from SNR loss. We propose a novel CS algorithm for dynamic CE-MRA that integrates magnitude subtraction into the reconstruction to avoid direct complex subtraction while taking advantages of the "subtraction sparsity".

## Methods

The reconstruction uses a split-Bregman minimization of the sum of the L1 norm of the pixel-wise magnitude difference between two successive temporal frames, i.e. |(|I_2 |-|I_1 |)|_1, a fidelity term and a total variation (TV) sparsity term.

Retrospective study: a full-sampled Cartesian 3D GRE sequence was used for the CE-MRA acquisitions on 6 volunteers with a resolution of 1x1x1.3-2.2 mm^3^. Datasets were retrospectively subsampled and different strategies of reconstruction were quantitatively and qualitatively evaluated: a) magnitude subtraction of the original k-space images; b) separate independent CS reconstructions (IDCS); c) k-space complex subtraction CS reconstruction (KDCS); d) magnitude subtraction CS reconstruction (MDCS).

Prospective study: a sequence was implemented that is capable of prospectively acquire under-sampled 3D dynamic CE-MRA data according to pre-defined sampling mask at 1x1x2.0 mm3 resolution. The net acceleration from random subsampling was set to 12-fold, enabling the acquisition of 6 volumes within a single breath-hold on 2 volunteers.

## Results

**Figure 1:**
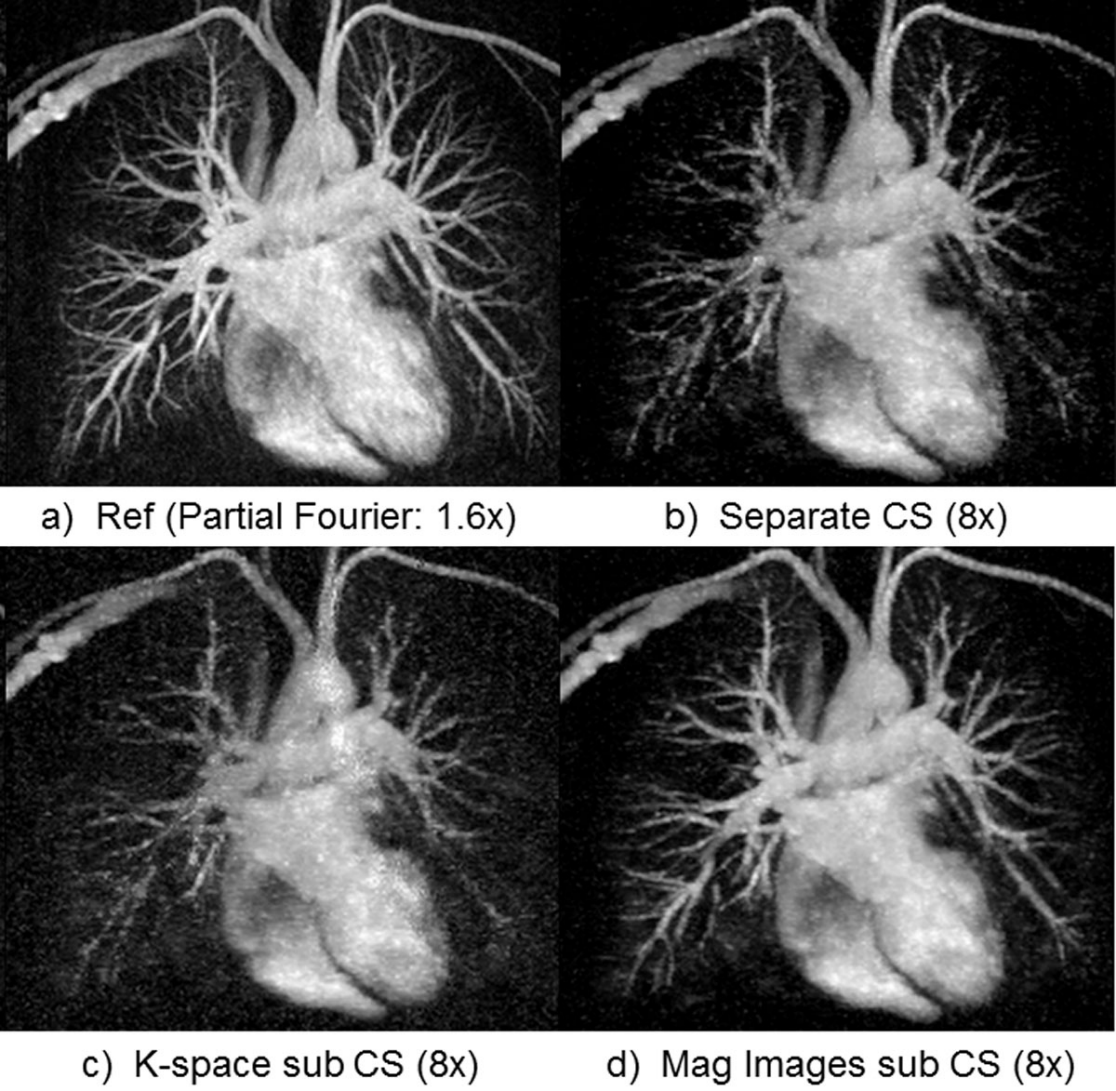
Thin MIP chest CE-MRA (zoomed, same windowing level). The proposed magnitude subtraction CS (d) reconstructs excellent image quality, close to reference (a) from highly under-sampled data while subtracted images from independent CS reconstruction (b) of each volume suffers from image degradation. Image quality is inferior using k-space subtraction CS reconstruction (c) due to significant SNR loss.

Proposed novel magnitude-subtraction CS reconstructs images with accurate details (Figure 1) compared to independent CS and less noise than complex-subtraction CS. At 10-fold acceleration, RMSE was lower for MDCS (20.36 %) compared to IDCS (28.93%, p<0.05) and KDCS (25.15%, p=0.07) and qualitative scores confirmed the superiority of MDCS images (2.59±0.51) to KDCS (1±0, p<0.01) and IDCS (1.75±0.45, p<0.05) images. K-space subtraction suffers very poor images quality due to the SNR loss from complex subtraction. Independent CS reconstruction has significantly lower images quality while magnitude subtraction CS images were considered "good" for all subsampling rates.

## Conclusions

**Figure 2:**
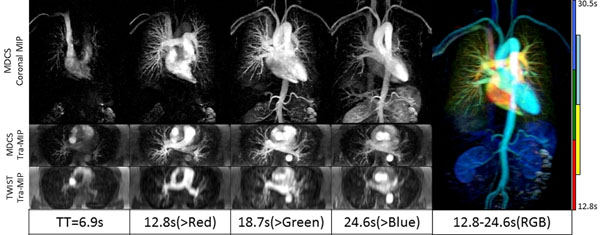
The high image quality of a prospectively subsampled (12.5-fold) dynamic high-resolution CS-CE-MRA allows obtaining both dynamic travel of contrast bolus in the circulatory system and clear delineation of small blood vessels. Thin MIP are reconstructed in coronal planes (after mask subtraction, 10cm thick) and transverse planes (without background subtraction, 6cm thick). The comparison with TWIST MIPs as used clinically in our institution shows the improvement of resolution in the slice direction (2mm instead of 6mm) at closest matching time points. The color-combined MIP (Right) provides both high-resolution vascular structure and dynamic information.

Based on our developed prospective CS dynamic CE-MRA sequence and our magnitude-subtraction based reconstruction algorithm, high quality dynamic CE-MRA is feasible even at 12.5x acceleration as shown in Figure 2.

CS-accelerated MRA has the potential to benefit MRA clinical practice. The extension of our algorithm with parallel imaging remains to be explored. The concept of magnitude subtraction can also be extended to many applications like dynamic contrast-enhanced MRI for perfusion imaging.

## Funding

The authors acknowledge research-funding support from American Heart Association (10SDG4200076), National Institutes of Health (1R21HL113427) and Siemens Healthcare.

